# Estimación de los límites de cambio *(deltacheck)* en el laboratorio clínico

**DOI:** 10.1515/almed-2021-0056

**Published:** 2021-07-21

**Authors:** Maria-José Castro-Castro, Lourdes Sánchez-Navarro

**Affiliations:** Laboratorio Clínico, Hospital Universitario de Bellvitge, Barcelona, Cataluña, España

**Keywords:** control de la plausibilidad, validación, autoverificación, límites de cambio, *deltacheck*

## Abstract

**Objetivos:**

Los límites de cambio, conocidos como *deltacheck*, son aquellos valores que indican sospecha de que la variación entre el resultado actual obtenido y el resultado anterior de la misma magnitud en un mismo paciente se debe a un error y, por tanto, dicho resultado ha de ser cuestionado. El propósito del presente estudio es establecer los límites de cambio para algunas magnitudes hematológicas y bioquímicas, con el fin de detectar resultados potencialmente erróneos, así como evaluar su eficacia a la hora de detectar resultados erróneos, para estandarizar el proceso de control de la plausibilidad.

**Métodos:**

Se calcularon los límites de cambio para 13 magnitudes bioquímicas y 6 hematológicas. Para cada magnitud, se calcularon las diferencias relativas (D), expresadas como la diferencia porcentual entre dos resultados consecutivos en el mismo paciente. A partir de dichas diferencias (D), se calcularon los percentiles 5 y 95 de la distribución de datos.

Para evaluar eficacia de los límites de cambio se emplearon 43 informes de laboratorio considerados erróneos a partir del procedimiento habitual de control de la plausibilidad utilizado en el laboratorio.

**Resultados:**

De los 43 informes de laboratorio que contenían algún error, 31 (72%) fueron clasificados como errores de contaminación por administración endovenosa y 12 (28%) como errores en la identificación del paciente. Todos los informes de laboratorio erróneos fueron detectados al aplicar conjuntamente los límites de cambio estimados de las diferentes magnitudes.

**Conclusiones:**

La mejor combinación de magnitudes en la misma muestra capaces de detectar informes de laboratorio erróneos fue: concentración de potasio, albúmina, creatinina, glucosa y hemoglobina.

## Introducción

El control de la plausibilidad, conocido como "proceso de validación", se define como el conjunto de procedimientos empleados para determinar si el resultado analítico de un paciente es válido, según una serie de criterios clínicos y biológicos predefinidos [1], [2], [3], [4], [5]. Representa el último proceso de la fase postanalítica, y se realiza con el objeto de garantizar la calidad de un informe de laboratorio clínico previamente a su envío al solicitante. Este procedimiento también es útil en el cribado de resultados erróneos debidos a errores en la toma de la muestra o en la introducción de datos, interferencias, etc. [6], [7], [8], [9], [10]. Los resultados identificados como satisfactorios en este proceso son considerados plausibles y aceptables, por lo que se pueden remitir al solicitante en un informe de laboratorio clínico.

Para el control de la plausibilidad se emplean diferentes herramientas, que detectan resultados sospechosos de ser erróneos, como los límites de alerta (intervalo de valores, que cuando se supera, indican un posible resultado erróneo) [11], límites de cambio (intervalo de valores de las diferencias obtenidas entre dos resultados consecutivos en el mismo paciente que, cuando se supera, indica un posible resultado erróneo) [11] y límites de predicción (intervalo de valores de una magnitud que se establece a partir de otra magnitud relacionada fisiopatológicamente que, cuando se supera, indica un posible error en el resultado) [12]. Si se superan los límites establecidos, se inicia un proceso para determinar si el resultado obtenido es correcto o no. Dicho proceso implica la verificación de la información sobre la muestra, el diagnóstico, la repetición de la medición, y la solicitud de obtención de una nueva muestra [13].

Los límites de cambio, conocidos como *deltacheck*, son aquellos que indican que la variación entre el resultado actual obtenido y el anterior en la misma magnitud y en el mismo paciente podría deberse a un error y, por tanto, dicho resultado debe ser cuestionado. En condiciones fisiológicas, la diferencia observada entre dos resultados en la misma magnitud y en el mismo paciente es el efecto combinado de la variabilidad preanalítica, analítica y biológica intraindividual. Esta diferencia variará según las circunstancias, pero de una medida "razonable" (esto es, el resultado obtenido concuerda con el anterior). Cuando existe una discrepancia entre un resultado y el anterior, éste se considera sospechoso de ser erróneo. Existen diversas estrategias para establecer límites de cambio, entre las que se incluyen datos subjetivos basados en la experiencia del personal del laboratorio, los médicos especialistas, o la literatura científica [14, 15]. Los límites de cambio también se pueden obtener a partir de datos objetivos empleando, entre otros, datos de la variabilidad biológica intraindividual (dentro de un mismo individuo) [13, 15] o percentiles de distribución poblacionales de las diferencias [11, 15], [16], [17], [18]. El intervalo de tiempo en el que se mide la variación es otro [aspecto a la hora de establecer estos límites. La mayor parte de los estudios publicados muestran un elevado grado de variación en el intervalo de tiempo seleccionado, probablemente debido a la falta de recomendaciones específicas y a la ausencia de evidencia científica. No obstante, los intervalos de tiempo para las magnitudes bioquímicas y hematológicas suelen ser cortos, oscilando entre 3 y 7 días [13].

El control de la plausibilidad se puede aplicar de forma estandarizada o no. El control de la plausibilidad no estandarizado lo realizan los profesionales de laboratorio mediante inspección visual de los resultados obtenidos. Este proceso tiene un elevado grado de subjetividad y variación interindividual, ya que los especialistas aplican su propio criterio, adoleciendo de una falta de estandarización en los algoritmos o criterios empleados. Por otro lado, el control de la plausibilidad informatizado permite la estandarización del proceso. En este tipo de control de la plausibilidad se aplican reglas, algoritmos y sofisticados sistemas para detectar resultados que superan los límites de alerta, los límites de cambio *(deltacheck)* o los límites de predicción. Este sistema identifica resultados que se consideran aceptables para el solicitante [1]. La automatización ofrece diversas ventajas, entre las que se encuentran la eliminación de la variación interindividual, la mejora en la eficiencia del proceso y el ahorro de tiempo y esfuerzo.

Aunque la mayoría de los laboratorios clínicos disponen de sistemas informáticos para estandarizar total o parcialmente el proceso de control de la plausibilidad, éste se suele realizar de forma no estandarizada. Los sistemas estandarizados se suelen emplear en laboratorios clínicos grandes con una gran carga de trabajo. Sin embargo, sólo entre el 25% y el 40% de los laboratorios que emplean sistemas de verificación, según la literatura, emplean *deltacheck* para detectar posibles errores [19, 20]. Las razones son diversas: el estudio de *deltacheck* y la verificación de su efectividad resultan costosos y laboriosos, por lo que existen pocas publicaciones que aporten límites de cambio verificados para ser configurados en los sistemas informáticos de los laboratorios. Por otro lado, no todos los sistemas informáticos de los laboratorios disponen de todas las ecuaciones que se emplean para la estimación de los límites de cambio. El propósito del presente estudio es establecer límites de cambio para algunas magnitudes hematológicas y bioquímicas para detectar resultados potencialmente erróneos, así como evaluar su eficacia para detectar resultados erróneos, con el objetivo final de estandarizar el proceso del control de la plausibilidad.

## Materiales y métodos

El estudio se realizó en el laboratorio clínico del Hospital Universitari de Bellvitge (Barcelona, España). El Hospital Universitari de Bellvitge es un hospital dotado de 900 camas especializado en pacientes adultos, que cuenta con numerosas especialidades médico-quirúrgicas, a excepción de pediatría y obstetricia. En él ingresan anualmente 35.000 pacientes, de los cuales, alrededor del 28,5% son pacientes de gran complejidad (oncología, cardiología, nefrología, gastroenterología…). Asimismo, se realizan anualmente unas 12.000 intervenciones de cirugía mayor y unos 180 trasplantes de órgano sólido. El laboratorio clínico cuenta con la Acreditación Nacional ISO 15189.

Para calcular los límites de cambio, se exportaron los resultados de solicitudes analíticas ordinarias de la base de datos del laboratorio clínico utilizando el programa Omnium (Roche Diagnostics), de los pacientes ingresados entre el 1 de enero de 2018 y el 31 de diciembre de 2018. Se eligió un periodo amplio de tiempo para conseguir el mayor número posible de pacientes de los que se dispusiera de resultados previos.

Se seleccionaron una serie de magnitudes bioquímicas y hematológicas. Esta selección se basó en su efectividad a la hora de detectar errores de identificación y contaminación, siguiendo las recomendaciones de la *CLSI: “Use of deltachecks in the medical laboratory”*(21).

Las magnitudes bioquímicas seleccionadas fueron: concentración de albúmina, alanina aminotransferasa, fosfatasa alcalina, aspartato aminotransferasa, bilirrubina, calcio (II), cloruro, creatinina, gamma-glutamiltransferasa, glucosa, ion potasio, ion sodio y urea.

Las magnitudes hematológicas seleccionadas fueron: concentración de número de eritrocitos, leucocitos y plaquetas; concentración de masa de hemoglobina; fracción de volumen de los eritrocitos (hematocrito) y volumen entítico de los eritrocitos (volumen corpuscular medio o VCM).

Las mediciones de las magnitudes bioquímicas se realizaron con el sistema Cobas c701 (Roche Diagnostics), mientras que las magnitudes hematológicas se midieron con el analizador Sysmex XN (Kobe, Japan).

Para calcular los límites de cambio, los resultados inferiores al límite de detección correspondiente se consideraron iguales al valor numérico de dicho límite. El intervalo de tiempo, entre los dos resultados consecutivos de un paciente, empleado para calcular los límites de *deltacheck* fue de un año. Se calculó el percentil 90 de días transcurridos entre los dos resultados consecutivos del mismo paciente.

Para cada magnitud, se calcularon las diferencias relativas (D), expresadas como el porcentaje de variación entre dos resultados consecutivos de un mismo paciente mediante la siguiente ecuación, teniendo en cuenta el resultado más reciente (resultado actual) y el resultado anterior:

$$D=\frac{\text{resultado actual}-\text{resultado anterior}}{\text{resultado anterior}}\times 100\left(\%\right)$$

Un valor negativo indica que, para la misma magnitud en el mismo paciente, el resultado actual es inferior al anterior, mientras que un valor positivo indica un incremento en el resultado actual con respecto al previo.

A partir de dichas diferencias (D), se calcularon los percentiles 5 y 95 de la distribución de datos. De este modo, se excluyó el 10% de los resultados, estableciendo el percentil 5 como límite de cambio inferior y el percentil 95 como límite de cambio superior.

Para evaluar la eficacia de los límites de cambio para detectar errores de laboratorio, se obtuvieron, a partir del procedimiento habitual de control de la plausibilidad del laboratorio y en el periodo comprendido entre enero y abril de 2018, 43 informes de laboratorio que contenían algún error. Ante la sospecha de error debido a contaminación por administración endovenosa o de error de identificación de la muestra, se había solicitado en su momento una segunda muestra. La consideración de informe erróneo se basó en la decisión final y subjetiva del personal del laboratorio al aplicar el procedimiento estándar de control de plausibilidad, tras comparar los resultados de la primera y la segunda muestra.

Se clasificaron dos tipos de error en los informes de laboratorio: contaminación por administración endovenosa (error analítico tipo 1) y errores de identificación de muestras (error analítico tipo 2). Se aplicaron los límites de cambio estimados a cada magnitud e informe de laboratorio y se calculó la capacidad de detectar errores de cada magnitud de forma individual, así como también de forma general cuando todos los límites de cambio se aplican de forma combinada. Asimismo, se evaluó la mejor combinación de magnitudes en la detección de informes erróneos: para ello, se fueron añadiendo sucesivamente las magnitudes con la mayor efectividad para detectar errores, hasta lograr la combinación que se identificó el mayor número de informes erróneos.

Todos los análisis estadísticos se realizaron con el programa SPSS v.17 (SPSS, Chicago, US)

## Resultados

En las Tablas 1 y 2 se muestran los límites de cambio de las magnitudes bioquímicas y hematológicas, respectivamente. En ambas tablas se describen las magnitudes utilizando la sintaxis recomendada por la Unión Internacional de Química Pura y Aplicada (IUPAC) y la Federación Internacional de Química Clínica y Medicina de Laboratorio [21]. Estas tablas muestran el intervalo de tiempo entre los dos resultados de un paciente (mediana con rango intercuartílico), el número de resultados obtenidos para cada magnitud (n), el número y fracción (%) de resultados para los que se disponía de un resultado previo del mismo paciente, y los límites de cambio (%). La mediana de días entre los dos resultados del mismo paciente fue de 3 días (con un rango intercuartílico de entre 4 y 5 días) y el percentil 90 fue inferior a los 18 días.

**Tabla 1:** Límites de variación de magnitudes bioquímicas de solicitud ordinaria.

Magnitud bioquímica (unidades)	n	Intervalo de tiempo de mediana (IQR)	n con resultados previos, %	Límite de cambio de *p5* inferior, %	Límite de cambio de *p95* superior, %
S; Aspartato aminotransferasa; c.cat, µkat/L	39558	3 (4)	23677 (60%)	−57	109
S; Albúmina; c. masa, mg/L	34715	3 (4)	19564 (56%)	−17	21
S; Fosfatasa alcalina; c.cat., µkat/L	32660	3 (5)	18449 (56%)	−39	38
S; Aspartato aminotransferasa; c.cat., µkat/L	29388	3 (5)	15294 (52%)	−54	144
S; Bilirrubina; c. sust., µmol/L	29830	3 (4)	15571 (52%)	−46	101
S; Calcio (II); c.sust., mmol/L	18088	3 (5)	8243 (46%)	−10	10
S; Creatinina; c.sust., µmol/L	42385	3 (4)	26570 (63%)	−25	40
S; Glucosa; c.sust, mmol/L.	41656	3 (4)	25841 (62%)	−37	68
S; γ-Glutamiltransferasa; c.cat., µkat/L	37424	3 (4)	21920 (559%)	−58	62
S; Iones de potasio; c.sust., mmol/L	39944	3 (4)	21887 (55%)	−19	22
S; Sodio; c.sust., mmol/L	42162	3 (4)	26309 (62%)	−4	4
S; Urea; c.sust., mmol/L	39289	3 (4)	24521 (62%)	−44	77

Intervalo de tiempo: mediana (RIC) = intervalo de tiempo en el que se evalúan dos resultados secuenciales de un mismo paciente para calcular sus límites de cambio, expresado en medianas y rangos intercuartílicos (RIC).

**Tabla 2:** Límites de variación de pruebas hematológicas de carácter ordinario.

Magnitud hematológica	n	Intervalo de tiempo de mediana (IQR)	n con resultados previos, n (%)	Límite de cambio de *p5* inferior, %	Límite de cambio de *p95* superior, %
B; Eritrocitos; conc.núm., 10^12^/L	40256	3 (4)	23890 (59%)	−18	20
B; Hemoglobina; c.masa, g/L	40447	3 (4)	24632 (61%)	−18	20
B; Eritrocitos; fr.vol., %	40447	3 (4)	24632 (61%)	−18	20
B; Eritrocitos; vol ent., fL	40449	3 (4)	24636 (61%)	−4	4
B; Leucocitos; conc., 10^9^/L	39938	3 (4)	23614 (59%)	−43	77
B; Plaquetas; nº c., 10^9^/L	40159	3 (4)	24056 (60%)	−43	60

Intervalo de tiempo: mediana (RIC) = intervalo de tiempo en el que se evalúan dos resultados secuenciales de un mismo paciente para calcular sus límites de cambio, expresado en medianas y rangos intercuartílicos (RIC).

De los 43 informes erróneos, 31 (72%) fueron considerados errores de contaminación por administración endovenosa y 12 (28%) como errores de identificación.

La Tabla 3 incluye el número y fracción del total de informes erróneos detectados para cada magnitud y para cada tipo de error.

**Tabla 3:** Porcentaje de errores detectados.

Magnitud (unidades)	n	Errores detectados, n (%)	Errores de tipo 1, n	Errores tipo 1 detectados, n (%)	Errores tipo 2, n	Errores tipo 2 detectados, n (%)
S; Iones de potasio; c.sust., mmol/L	42	27 (64)	30	21 (70)	12	6 (50)
S; Albúmina; c. masa, mg/L	23	14 (61)	18	9 (50)	5	5 (100)
S; Creatinina; c.sust., µmol/L	43	25 (58)	31	15 (48)	12	10 (83)
S; Glucosa; c.sust, mmol/L.	43	24 (56)	31	21 (68)	12	3 (25)
B; Hemoglobina; c.masa, g/L	42	23 (55)	30	17 (57)	12	6 (50)
B; Eritrocitos; conc., 10^12^/L	42	23 (55)	30	17 (57)	12	6 (50)
S; Sodio; c.sust., mmol/L	43	22 (51)	31	17 (55)	12	5 (42)
B; Eritrocitos; fr.vol., %	42	21 (50)	30	15 (50)	12	6 (50)
S; Urea; c.sust., mmol/L S; γ-	32	14 (44)	24	8 (33)	8	6 (75)
S; Bilirrubina; c.sust., µmol/L	28	11 (39)	22	7 (32)	6	4 (67)
S; Calcio (II); c.sust., mmol/L	19	7 (37)	14	5 (36)	5	2 (40)
B; Leucocitos; conc., 10^9^/L	42	15 (36)	30	10 (33)	12	5 (42)
S; Fosfatasa alcalina; c.cat., µkat/L	25	6 (24)	19	5 (26)	6	1 (17)
B; Eritrocitos; vol ent., fL	42	10 (24)	30	6 (20)	12	4 (33)
S; Aspartato aminotransferasa; c.cat., µkat/L	36	8 (22)	26	4 (15)	10	4 (40)
S; Aspartato aminotransferasa; c.cat., µkat/L	27	4 (15)	21	3 (14)	6	1 (17)
B; Plaquetas; nº c., 10^9^/L	42	6 (14)	30	3 (10)	12	3 (25)
S; -Glutamiltransferasa; c.cat., µkat/L	33	4 (12)	25	4 (16)	8	0 (0)

Errores tipo 1, errores de contaminación por administración endovenosa; errores tipo 2, errores de identificación.

Todos los informes erróneos de laboratorio fueron detectados al aplicar conjuntamente los límites de cambio estimados.

La combinación de las magnitudes con mayor capacidad de detección de error fue: concentración de potasio, creatinina, albúmina, glucosa y hemoglobina.

**Figura 1:**
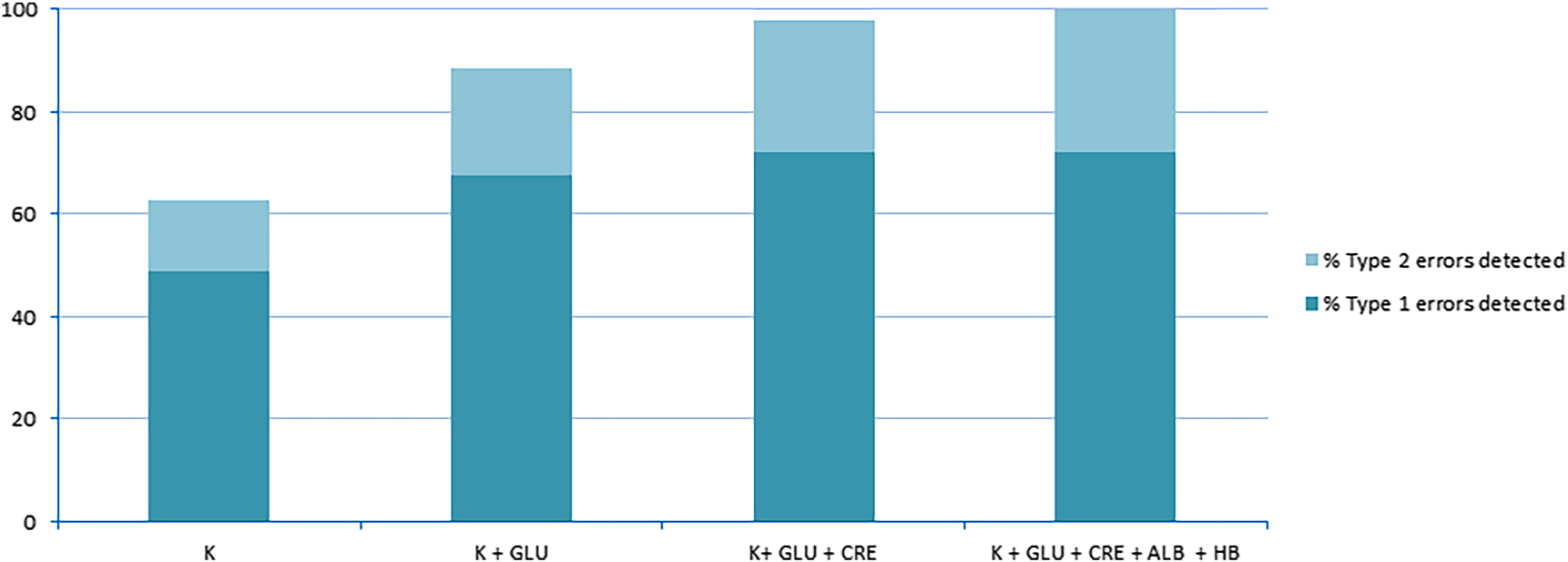
Porcentaje de errores detectados por diferentes combinaciones de magnitudes. K, concentración de ion potasio en suero; GLU, concentración de glucosa en suero; CRE, concentración de creatinina en suero; ALB, concentración de albúmina en suero; HB, concentración de masa de hemoglobina. Errores de tipo 1, errores de administración endovenosa; errores tipo 2, errores de etiquetado.

En la Figura 1 se muestra el porcentaje de errores detectados por las diferentes combinaciones de magnitudes.

## Discusión

En el control de la plausibilidad, el límite de cambio es la herramienta más importante para detectar errores de laboratorio (esto es, contaminación de muestras por administración intravenosa o errores en la identificación de las mismas).

A la hora de establecer los límites de cambio, se deben tener en cuenta los siguientes factores: la selección de las magnitudes de mayor utilidad, el origen de los límites de cambio (percentiles de diferencias en la distribución, valor de la referencia del cambio…), el origen de la población seleccionada (pacientes ambulatorios, ingresados…), la selección de los intervalos de tiempo entre resultados consecutivos y la evaluación de la efectividad de los límites estimados.

En este estudio, las magnitudes se seleccionaron en base al documento de la CLSI *“Use of deltachecks in the medical laboratory”* [19], en donde se establecen los criterios para seleccionar las magnitudes más útiles a la hora de detectar los errores más frecuentes en el laboratorio, basándose en la variabilidad interindividual e interindividual y el índice de individualidad [22]. Según el documento, es preferible seleccionar magnitudes con baja variabilidad individual como el volumen entítico de los eritrocitos (VCM). Un índice de individualidad <0,60 indica que la magnitud es útil para detectar la identificación incorrecta del espécimen.

Se pueden aplicar diferentes estrategias para establecer los límites de cambio. El modelo seleccionado se ha basado en el uso de percentiles de distribución a partir de datos del laboratorio. Estos límites incluyen la variabilidad asociada a la imprecisión y sesgo de los sistemas de medida, así como a la variabilidad biológica y patológica basada en las características de la población (pacientes ambulatorios o ingresados) [11]. Este modelo basado en la distribución de los valores delta de la población está descrito en el documento del Instituto de Estándares Clínicos y de Laboratorio [19].

Con respecto al método de cálculo, la fórmula empleada para calcular los límites de cambio depende directamente de las disponibles en el sistema de información del laboratorio. En este modelo, los límites de cambio configurables en el sistema informático Infinity (Roche Diagnostics) se podían expresar en diferencias relativas o valores absolutos.

Asimismo, cada sistema informático puede calcular estas diferencias relativas de forma diferente, y los límites de cambio establecidos a partir de otras ecuaciones pueden no ser válidos. En una publicación anterior, se empleó una ecuación basada en el resultado más alto y más bajo obtenido en dos mediciones consecutivas en el mismo paciente [11].

$$D=\frac{\text{Resultado más alto}-\text{resultado más bajo}}{\text{Resultado más bajo}}\times 100$$

Este estudio se realizó para adaptar los límites de cambio a las ecuaciones disponibles y mostrar el desarrollo de nuestro modelo. Se calcularon los límites de cambio de numerosas magnitudes bioquímicas, observándose que dichos límites diferían según origen de la muestra (ordinaria o de carácter urgente). En general, los límites de cambio estimados a partir de solicitudes urgentes deberían ser más amplios que los obtenidos a partir de solicitudes ordinarias.

Las Tablas 1 y 2 muestran que las magnitudes bioquímicas y hematológicas con un intervalo de referencia corto y un elevado control homeostático tienen límites de cambio más bajos. Por ejemplo, los límites de cambio obtenidos fueron inferiores a ±10% para la concentración de sodio, calcio (II) y para el volumen de los eritrocitos. Los límites de cambio obtenidos para cada magnitud suelen ser superiores a los indicados en la literatura. Dos publicaciones recientes [23, 24] revelan las siguientes variaciones: 3,2 y 4,4% para la concentración de ión sodio; 6,2 y 7,8% y para la de calcio; 9,7; 11,5% para la de concentración de albúmina; 18,3 y 20,8% para la de creatinina; 18,8 y 23% para de la fosfatasa alcalina, así como 16 y 16,7% para la de glucosa. Estos límites proceden de datos obtenidos de la variación biológica, en la que sólo se incluye la variabilidad fisiológica. Los límites de cambio expresados en percentiles también incluyen la variabilidad patológica e iatrogénica, con lo que han de ser superiores, siendo especialmente importantes cuando se aplica el control de la plausibilidad a pacientes ingresados. Esto es relevante en el caso de la medición de glucosa, ya que su concentración está directamente relacionada con un procedimiento tan habitual como es su administración intravenosa. De esta forma, los límites de cambio expresados en percentiles evitarán la identificación de multitud de informes identificados como sospechosos de contener errores tras una inspección minuciosa (falsos positivos).

Con especto a los intervalos de tiempo para establecer los límites de cambio, el 90% de los datos utilizados para su estimación presentaban un intervalo inferior a los 18 días. Aunque no existen recomendaciones claras, los intervalos de tiempo obtenidos son, al parecer, relativamente cortos.

Los límites de cambio establecidos en este estudio identificarán como potencialmente erróneos el 10% de los resultados de cada magnitud. Este porcentaje se seleccionó de forma arbitraria, considerando los profesionales de laboratorio disponibles para poder revisar los resultados. La selección de los percentiles adecuados depende de las necesidades de cada laboratorio y de su eficacia para detectar errores en los especímenes. Existen escasas publicaciones sobre la validación o verificación de la efectividad de los límites de cambio. La mayoría se basan en simulaciones de errores, en las que el laboratorio identifica intencionadamente una muestra de forma errónea, contamina o altera de alguna forma los especímenes. En nuestro estudio, los errores fueron reales, basados en la decisión final de los profesionales de laboratorio tras aplicar el procedimiento ordinario de control de la plausibilidad. Los límites de cambio estimados debían ser, como mínimo, tan eficaces como el sistema de control de la plausibilidad vigente en el laboratorio.

En este estudio solamente se han contemplado los tipos de errores más frecuentes en el laboratorio clínico (contaminación por administración endovenosa y errores de identificación). No se contemplaron otros tipos de errores, puesto que estos se detectan con otras herramientas. Las muestras hemolizadas, por ejemplo, se detectan a partir de la medición del índice de hemólisis de la muestra y, en las magnitudes afectadas, se sustituye el resultado por el comentario de "muestra hemolizada". Las muestras coaguladas se detectan mediante inspección visual o a partir de resultados que exceden los límites de alerta. Otros tipos de errores, como la interferencia debido a la administración de fármacos, son menos frecuentes en el laboratorio. No obstante, serían necesarios más estudios sobre la detección de estos tipos de errores.

La Tabla 3 y la Figura 1 revelan que las mejores magnitudes para detectar errores de laboratorio son: concentración de potasio, albúmina, creatinina, glucosa, hemoglobina, eritrocitos y sodio. Para cada tipo de error de laboratorio, las mejores magnitudes para detectar contaminación por administración endovenosa fueron: concentración de potasio, creatinina, glucosa, hemoglobina, eritrocitos, sodio y fracción de volumen de los eritrocitos (hematocrito). Las mejores magnitudes para detectar errores de identificación fueron: concentración de albúmina, creatinina, urea y bilirrubina. Ninguna magnitud por sí sola se mostró eficaz para detectar la totalidad de informes erróneos. La concentración de potasio, a pesar de ser la mejor magnitud para detectar errores, solamente detectó el 64% de ellos. Como sabemos, un informe de laboratorio muestra los resultados para una serie de magnitudes, y la aplicación conjunta de límites de cambio a diferentes magnitudes aumenta su efectividad a la hora de detectar errores. En la Figura 1 se muestra que todos los informes erróneos se pueden detectar mediante el uso combinado de algunas magnitudes, cuyo análisis se suele pedir rutinariamente a los laboratorios. La mejor combinación de magnitudes es: concentración de potasio, albúmina, creatinina, glucosa y hemoglobina. Sin embargo, para lograrlo, la medición de estas magnitudes debe realizarse simultáneamente.

Los límites de cambio no siempre son aplicables, debido principalmente a la ausencia de resultados previos o a un intervalo de tiempo entre dos resultados consecutivos que supera el límite establecido. En nuestro estudio, teniendo en cuenta que los datos recogidos correspondían a pacientes ingresados, solo había resultados previos en el intervalo para el 46–63% de los pacientes (no se tuvieron en cuenta las peticiones urgentes para el mismo paciente). Si se establecen límites de cambio para pacientes ambulatorios, es previsible que la proporción de pacientes con resultados previos disponibles sea menor, factor a tener en cuenta a la hora de implementarlos. En estos casos, para detectar un resultado sospechoso, son decisivas otras herramientas de plausibilidad como los límites de alerta o predicción. Por otro lado, los facultativos no siempre solicitan el análisis de las mismas magnitudes, por lo que la efectividad de los límites de cambio dependerá de las magnitudes solicitadas.

Los resultados obtenidos ejemplifican la implementación de los límites de cambio en un laboratorio clínico y demuestran que cada laboratorio debería calcular sus propios límites y personalizar la configuración en función del sistema informático empleado. También se ofrecen límites de cambio para su adopción por otros laboratorios clínicos, previa verificación en la población de pacientes del laboratorio clínico.

Con respecto al tiempo transcurrido para calcular y aplicar los límites de cambio, no existe evidencia sobre el tiempo óptimo para aplicar dichos límites, y no existen recomendaciones al respecto [25, 26].

Lee et al. indican que los límites de cambio basados en percentiles proporcionan una distribución simétrica para algunas de las magnitudes (esto es, concentración de sodio, potasio, proteínas y albúmina), en concordancia con los resultados de este estudio [20]. Recientemente, se ha mostrado que cuanto mayor sea el número de magnitudes empleadas para la aplicación los límites de cambio, mayor será la sensibilidad de estos para detectar informes erróneos. Sin embargo, con este método, también puede aumentar el número de falsos positivos [27].

Una limitación de este estudio fue que no se calculó el porcentaje de falsos positivos en la detección de informes de laboratorio erróneos. Los límites de cambio sólo se aplicaron a los informes considerados erróneos. Para determinar si un informe es correcto o no, es necesario realizar una serie de procesos que pueden causar molestias al paciente, como obtener una nueva muestra. Teniendo en cuenta esta limitación, el criterio utilizado para establecer los límites fue la disponibilidad de personal para revisar los resultados analíticos sospechosos de ser erróneos. Por otro lado, se debería realizar este estudio combinando los resultados de las peticiones urgentes, así como de las ordinarias.

## Conclusiones

En este estudio se ofrecen los límites de cambio obtenidos en un laboratorio clínico con un elevado volumen de muestras, en el que la automatización y estandarización del control de la plausibilidad resultan fundamentales. Con esta herramienta, los laboratorios clínicos pueden estandarizar el proceso de control de la plausibilidad y minimizar la variabilidad intraindividual derivada de la subjetividad. De la misma forma, se reduciría el tiempo y el esfuerzo invertido en la revisión manual de todos los resultados sospechosos de ser erróneos.
